# Supplementary material for: Implications of Hyperoxia over the Tumor Microenvironment: An Overview Highlighting the Importance of the Immune System
Source: Cancers (Basel). 2022 May 31;14(11):2740. doi: 10.3390/cancers14112740 (PMC9179641; doi:10.3390/cancers14112740)
Supplement: Supplementary file 1 [file cancers-14-02740-s001.zip › Supplementary File S1.pdf]

cancers MDPI

File S1: Material and Methods: mRNA-seq analysis

1. Next-generation transcriptome sequencing (RNA-Seq) for gene expression analysis

Four different group of samples defined as "MUM2B Control", "HBEC3-KT Control", "HBEC3-KT Control Hyperoxia" and "MUM2B Control Hyperoxia", with three independent biological replicate experiments each, were mRNA extracted using RNeasy Plus Mini Kit (QIAGEN) and quantified using Nanodrop One (ThermoFisher).

Afterwards, samples were analysed to determine their RNA Integrity Number (RIN) coefficients using Bioanalyzer RNA 6000 Nano chip analysed by Bioanalyzer 2100 (Agilent Technologies Inc. CA, USA). Concentration of extracted RNA were obtained using the Qubit Fluorometer (Thermo Fisher Scientific, MA, USA). Then, libraries from mRNA were prepared using 1µg of RNA starting material and the TruSeq Stranded mRNA Library Prep Kit (Illumina, CA, USA) according to the manufacturer's protocol. Quality and size distribution of indexed mRNA libraries were validated through Bioanalyzer High Sensitivity DNA assay in a Bioanalyzer 2100 and concentration was measured on the Qubit fluorometer. Finally, libraries were pooled in an equimolecular manner and then diluted and denatured as recommended. The 40x2nt paired-end sequencing was conducted on a NextSeq 500 sequencer (Illumina, CA, USA) using the highest output mode producing 19 M raw paired reads sample on average.

2. Data Analysis

Transcriptomic samples were analysed using miARma-Seq pipeline [1], [2]. This workflow performs all steps from raw data to differential expressed genes (DEG) calculation. Firstly, raw data were evaluated using FastQC software to analysed the quality of the reads [3]. Subsequently, after sample filtering and balancing the number of reads per sample, we obtained a mean of 52% in GC content and an average of 15,991,855 reads per sample. No adapter accumulation and bad quality reads (q<30) were found.

Afterwards, miARma-Seq aligns all sequences using STAR [4], resulting in an 84.97% of properly aligned reads. With this aim, Homo sapiens Gencode version v34 genome-build: GRCh38 was used as reference genome. After that, featureCounts software [5] was used to assign sequence reads to genes by using reference gene annotation (obtained from Gencode from the same assembly and genome build).

3. Differential expression

To perform the differential expression analysis, edgeR package was used [6]. Low expressed genes were removed and remaining genes were normalized by the trimmed mean of M-values (TMM) method [7]. Counts per million (CPM) and log2-counts per million (log-CPM) were used for exploratory plots [6] to check the consistency of the replicates. Furthermore, reads per kilo base per million mapped reads (RPKM), was calculated per gene on each sample.

In order to infer the replicability of the samples, Principal component analysis (PCA) and Hierarchical Clustering of normalized samples were used to get a general overview on the similarity of RNA-sequencing samples [8], [9].

Differentially expressed genes (DEG) were calculated between MUM2B Control Normoxia and MUM2B Control Hyperoxia; and HBEC3-KT Control Normoxia vs HBEC3-KT Control Hyperoxia. All genes having a False discovery rate (FDR) value <0.05 were marked as DEG. log2FC (log 2 of fold change) were used to evaluate the significance and the change in expression of a gene respectively between both types of samples.

4. Enrichment analysis Discussion

In order to identify the effects of differential gene expression, functional enrichment study was carried out using the clusterProfiler Bioconductor package [10]. To this end, DEG were compared against all expressed genes in the RNA-seq assay and we obtained

Gene ontology terms from the Bioconductor Homo sapiens database and associated to Entrez gene identifiers in an orgDB R object through the AnnotationForge package to be used with clusterProfiler. Therefore, Gene Ontology enrichment analysis was assessed for Biological Process, Molecular function and Cellular complex ontology terms. Furthermore, KEGG enrichment was also calculated using clusterProfiler, the gene names from our orgDB database and the "hsa" organism code from the KEGG database.

References

1. Andrés-León, E.; Núñez-Torres, R.; Rojas, A.M. miARma-Seq: A comprehensive tool for miRNA, mRNA and circRNA analysis. Sci. Rep. 2016, 6, 25749. https://doi.org/10.1038/srep25749.
2. Andrés-León, E.; Rojas, A.M. miARma-Seq, a comprehensive pipeline for the simultaneous study and integration of miRNA and mRNA expression data. Methods 2019, 152, 31–40. https://doi.org/10.1016/j.ymeth.2018.09.002.
3. Babraham Bioinformatics-FastQC a Quality Control Tool for High throughput Sequence Data. Available online: https://www.bioinformatics.babraham.ac.uk/projects/fastqc/ (accessed on 27 April 2022).
4. Dobin, A.; Davis, C.A.; Schlesinger, F.; Drenkow, J.; Zaleski, C.; Jha, S.; Batut, P.; Chaisson, M.; Gingeras, T.R. STAR: Ultrafast universal RNA-seq aligner. Bioinformatics 2013, 29, 15–21. https://doi.org/10.1093/bioinformatics/bts635.
5. Liao, Y.; Smyth, G.K.; Shi, W. feature Counts: An efficient general purpose program for assigning sequence reads to genomic features. Bioinformatics 2014, 30, 923–930. https://doi.org/10.1093/bioinformatics/btt656.
6. Nikolayeva, O.; Robinson, M.D. EdgeR for differential rna-seq and ChIP-seq analysis: An application to stem cell biology. In Stem Cell Transcriptional Networks: Methods and Protocols; Kidder, B.L., Ed.; Humana Press: New York, NY, USA, 2014; pp. 45–79. https://doi.org/10.1007/978-1-4939-0512-6_3.
7. Robinson, M.D.; Oshlack, A. A scaling normalization method for differential expression analysis of RNA-seq data. Genome Biol. 2010, 11, R25. https://doi.org/10.1186/gb-2010-11-3-r25.
8. Reeb, P.D.; Bramardi, S.J.; Steibel, J.P. Assessing Dissimilarity Measures for Sample-Based Hierarchical Clustering of RNA Sequencing Data Using Plasmode Datasets. PLoS ONE 2015, 10, e0132310. https://doi.org/10.1371/journal.pone.0132310.
9. Ritchie, M.E.; Belinda, P.; Wu, D.; Hu, Y.; Law, C.W.; Shi, W.; Smyth, G.K. limma powers differential expression analyses for RNA-sequencing and microarray studies. Nucleic Acids Res. 2015, 43, e47. https://doi.org/10.1093/nar/gkv007. PMID: 25605792; PMCID: PMC4402510.
10. Yu, G.; Wang, L.-G.; Han, Y.; He, Q.-Y. clusterProfiler: An R Package for Comparing Biological Themes Among Gene Clusters. OMICS J. Integr. Biol. 2012, 16, 284–287. https://doi.org/10.1089/omi.2011.0118.
