# Supplementary figures and images for: Implications of Hyperoxia over the Tumor Microenvironment: An Overview Highlighting the Importance of the Immune System
Source: Cancers (Basel). 2022 May 31;14(11):2740. doi: 10.3390/cancers14112740 (PMC9179641; doi:10.3390/cancers14112740)

## Slide 1
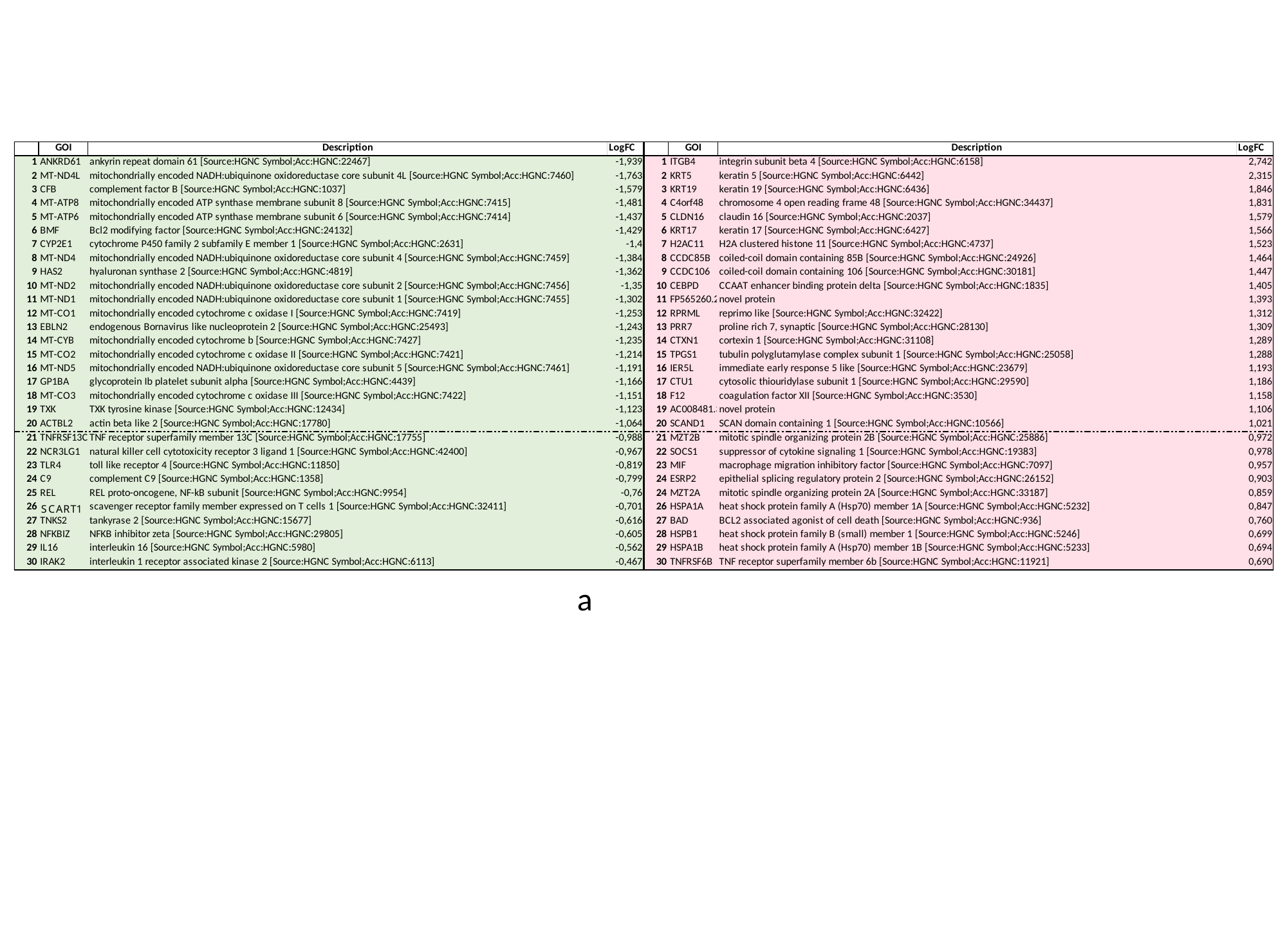

a

## Slide 2
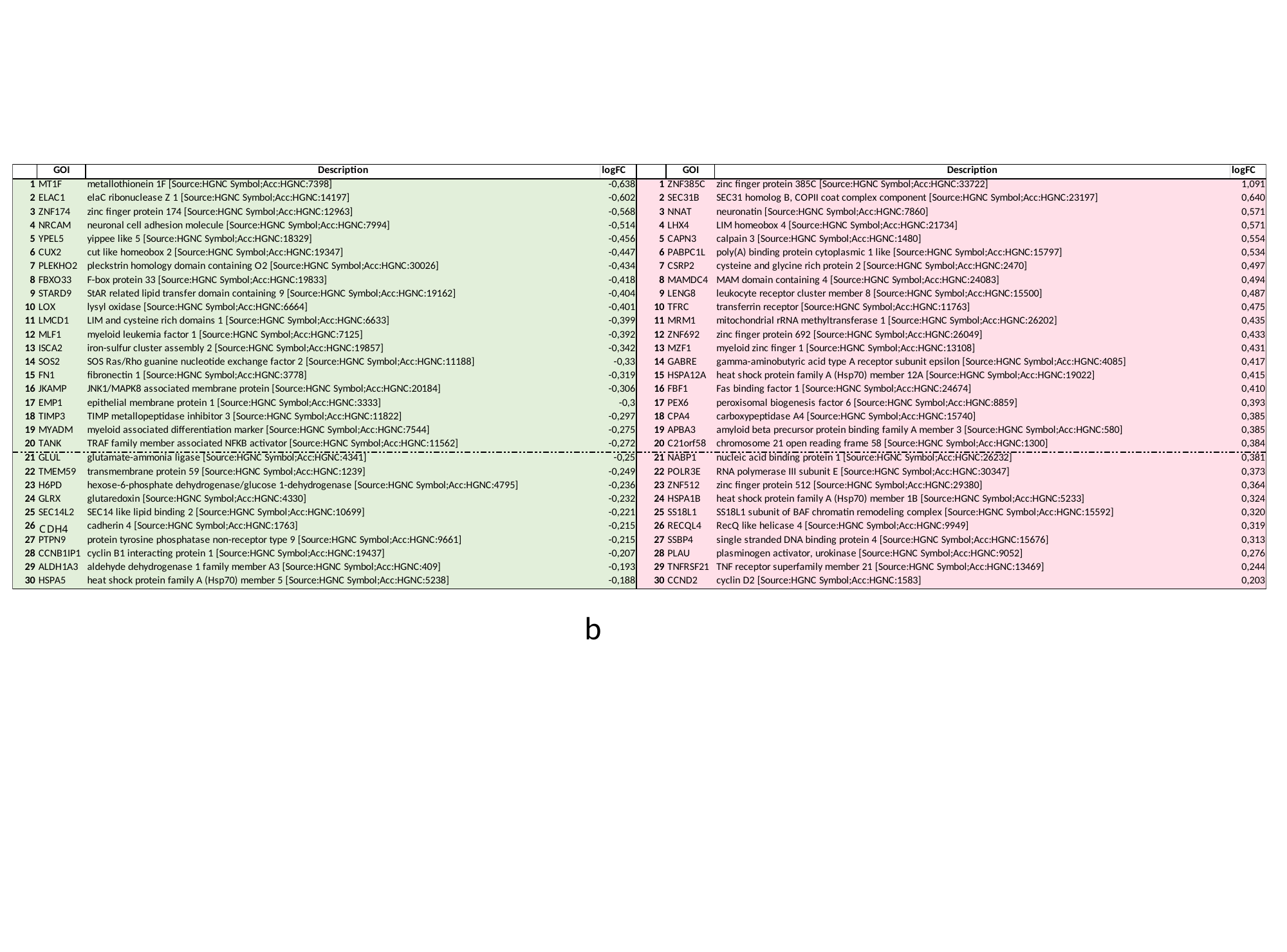

b

Supplement: Supplementary file 1 [file cancers-14-02740-s001.zip › Supplementary Figure S1.pptx]
